# MicroRNA Techniques: Valuable Tools for Agronomic Trait Analyses and Breeding in Rice

**DOI:** 10.3389/fpls.2021.744357

**Published:** 2021-09-20

**Authors:** Jiwei Chen, Sachin Teotia, Ting Lan, Guiliang Tang

**Affiliations:** ^1^Guangdong Provincial Key Laboratory for Plant Epigenetics, Longhua Bioindustry and Innovation Research Institute, College of Life Sciences and Oceanography, Shenzhen University, Shenzhen, China; ^2^Key Laboratory of Optoelectronic Devices and Systems of Ministry of Education and Guangdong Province, College of Optoelectronic Engineering, Shenzhen University, Shenzhen, China; ^3^Department of Biotechnology, Sharda University, Greater Noida, India; ^4^Department of Biological Sciences, Life Science and Technology Institute, Michigan Technological University, Houghton, MI, United States

**Keywords:** microRNA, short tandem target mimic, agronomic traits, crop breeding, CRISPR/Cas9

## Abstract

MicroRNAs (miRNAs) are a class of small noncoding RNAs that regulate gene expression at the post-transcriptional level. Extensive studies have revealed that miRNAs have critical functions in plant growth, development, and stress responses and may provide valuable genetic resources for plant breeding research. We herein reviewed the development, mechanisms, and characteristics of miRNA techniques while highlighting widely used approaches, namely, the short tandem target mimic (STTM) approach. We described STTM-based advances in plant science, especially in the model crop rice, and introduced the CRISPR-based transgene-free crop breeding. Finally, we discussed the challenges and unique opportunities related to combining STTM and CRISPR technology for crop improvement and agriculture.

## Introduction

MicroRNAs (miRNAs) are a class of short noncoding RNAs (20–24nt) that mediate gene expression by complementarily binding to their targeted transcripts for mRNA cleavage or protein translation inhibition (Sanei and Chen, 2015). Mature miRNAs are generated from precursor stem-loop structures (pre-miRNAs), which are the intermediates processed from primary miRNA transcripts (pri-miRNAs) transcribed by RNA polymerase II from *MIR* genes (Yu et al., 2019). Substantial evidence has shown that miRNAs play crucial roles in diverse biological processes, including plant development and biotic and abiotic stress responses. A growing number of yield-related agronomic traits have also been found to be associated with miRNAs (Zheng and Qu, 2015; Peng et al., 2019), which makes miRNAs promising targets for crop improvement.

**Figure 1:**
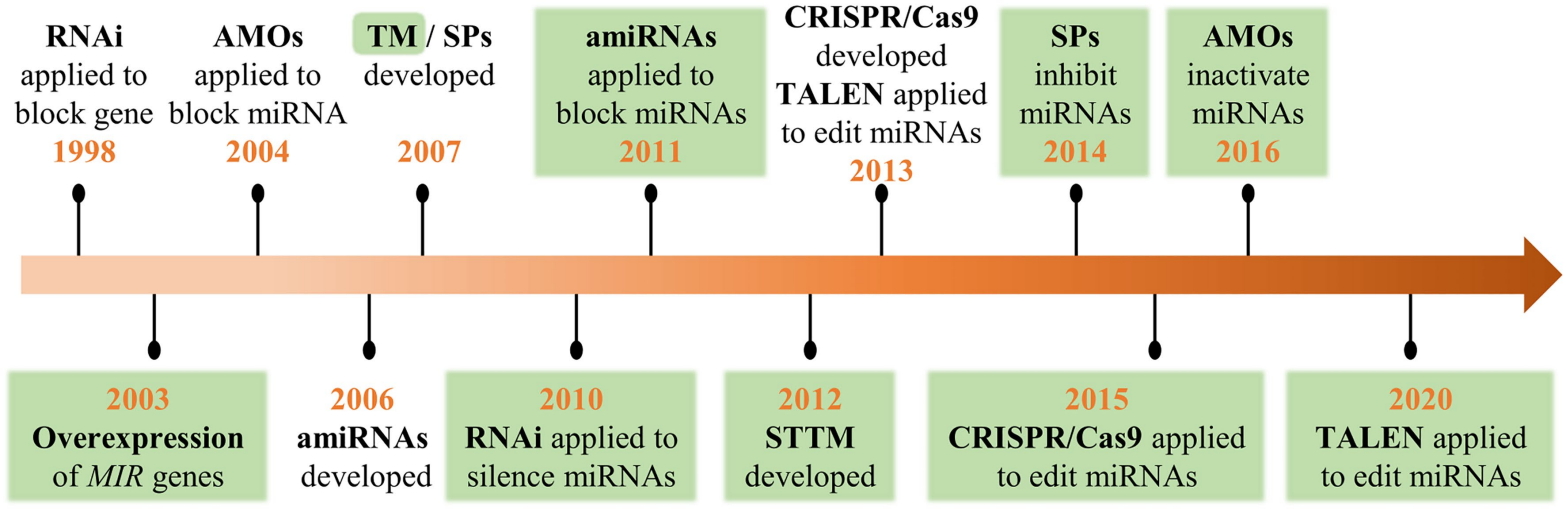
A historical timeline of the crucial technological developments in the functional analysis of genes and microRNAs (miRNAs). Green areas highlight the first application for plant miRNAs.

Since the first set of plant miRNAs was identified in *Arabidopsis* in 2002 (Llave et al., 2002; Park et al., 2002; Reinhart et al., 2002), an increasing number of miRNAs have been discovered and annotated due to high-throughput sequencing. However, the biological functions of most miRNAs have not been thoroughly explored. To decipher the function of miRNAs, multiple molecular-based approaches have been applied, such as the overexpression of *MIR* genes (Aukerman and Sakai, 2003), anti-microRNA oligonucleotides (AMOs; Hutvágner et al., 2004), RNA interference (RNAi; Vaistij et al., 2010), artificial miRNA (amiRNA; Eamens et al., 2011), endogenous and artificial target mimicry (Ebert et al., 2007; Franco-Zorrilla et al., 2007; Yan et al., 2012), transcription activator-like effector nucleases (TALEN; Hu et al., 2013), and clustered regularly interspaced short palindromic repeats/CRISPR-associated nuclease 9 (CRISPR/Cas9; Jacobs et al., 2015; Figure 1). Functional analyses of miRNAs have been achieved through overexpression for gain-of-function or knockdown/knockout for loss-of-function. Among these techniques, RNAi, amiRNA, TALEN, and CRISPR/Cas9, which were initially applied for the functional analysis of protein-encoding genes, have proven to be useful for the subsequent regulation of miRNAs (Fire et al., 1998; Schwab et al., 2006; Morbitzer et al., 2010; Shan et al., 2013). AMOs and sponges (SPs) were initially developed to inhibit miRNA action in animal systems and subsequently adopted in plants (Xian et al., 2014; He et al., 2016). MiRNA decoys/mimics such as target mimic (TMs) and short tandem target mimics (STTMs) were developed based on the discovery of miRNA sequestration by endogenous target mimics in plants (Franco-Zorrilla et al., 2007; Todesco et al., 2010; Yan et al., 2012).

These techniques have greatly expanded our abilities in plant miRNA research; more importantly, these methods are constantly being improved for broader applications in research and plant breeding. Thus, there is a strong need for a comprehensive understanding of the key features of each technique and effective controls on these methods for various research purposes. Here, we reviewed the major approaches used for determining the function of miRNAs, with a focus on the STTM technique and its applications in the functional characterization of miRNAs involved in various rice agronomic traits, and illustrated transgene-free breeding practices based on transgenic outcomes *via* CRISPR/Cas9. We also discussed the challenges and potential future trends of STTM applications in the functional analysis of miRNAs and in crop breeding.

## Techniques for Determining MiRNA Functions in Plants

### Gain-of-Function Analysis Techniques

**Figure 2:**
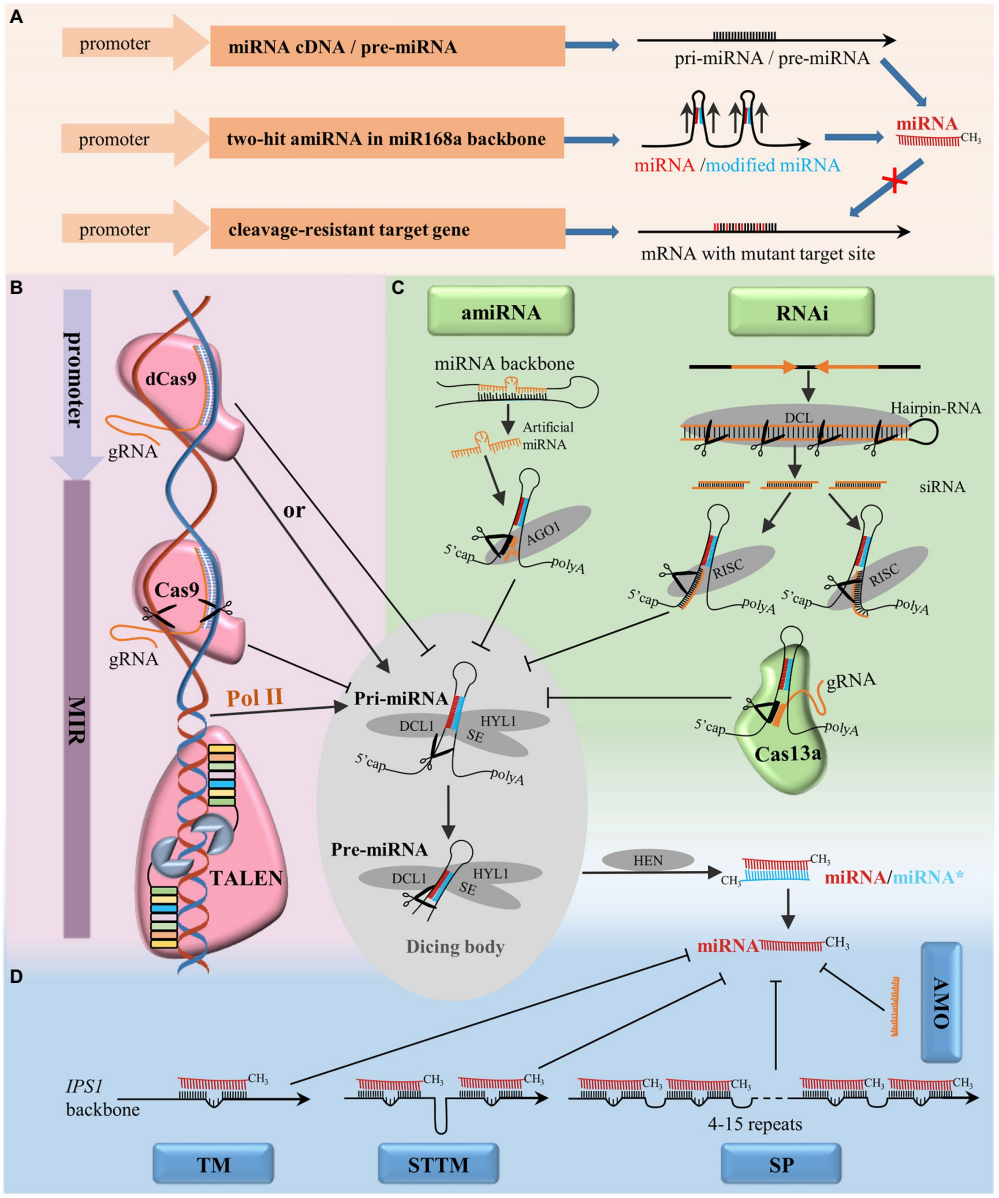
Techniques for the functional analysis of miRNAs in plants. **(A)** Transgenesis strategies to identify the functions of miRNAs *via* gain-of-function, overexpression of miRNAs by pre-miRNA, cDNA of miRNA or amiRNA, overexpression of miRNA cleavage-resistant target genes to mimic the effect of downregulating the expression of miRNA. **(B)** Techniques targeting miRNA genes: CRISPR-associated nuclease 9 (CRISPR/Cas9) system and TALEN for miRNA gene editing, CRISPR/dCas9 system for transcriptional regulation by targeting the promoter region. **(C)** Techniques targeting pri-/pre-miRNA: RNAi, amiRNA, and the CRISPR/Cas13a system. **(D)** Techniques targeting mature miRNA: AMO, TM, short tandem target mimic (STTM), and SP. AGO1, ARGONAUTE1; DCL1, DICER-LIKE1; HEN1, HUA ENHANCER1; HYL1, HYPONASTIC LEAVES1; RISC: RNA-induced silencing complex; SE, SERRATE.

There are several approaches to functionally characterize miRNAs based on gain-of-function strategies (Figure 2A). Both precursor miRNAs (pre-miRNAs; Boualem et al., 2008) and the full-length cDNA of *MIR* genes (Yang et al., 2013) can be used for miRNA overexpression. In addition, a vector system containing two-hit artificial miRNA in *Arabidopsis* miR168a backbone can also successfully overexpress endogenous miRNAs. In this approach, endogenous miRNAs are introduced by the insertion of complementary endogenous miRNA* with mismatches at positions corresponding to site 1 and site 12 of the miRNA strand (Ji et al., 2011). A strong constitutive 35S promoter is widely used to achieve gain-of-function effects, but conclusions derived from this strategy should be evaluated cautiously due to the misrepresentative expression level and pattern of miRNAs *in vivo*. In addition, given the involvement of miRNAs in diverse and complex regulatory networks, different transgenesis strategies should be adopted by exploiting specific promoters, such as tissue-specific, stress-induced, or developmental stage-specific promoters that correspond to specific research goals and can avoid pleiotropic effects (Peng et al., 2018).

### Loss-of-Function Analysis Techniques

#### Techniques Targeting the Genome Sequences of miRNAs

In recent years, several gene-editing tools, such as zinc-finger nucleases (ZFNs), TALENs, and CRISPR/Cas systems, have been developed based on different sequence-specific engineered endonucleases (SSNs), and some of them have been adopted for functional analysis of miRNAs in plants (Figure 2B). ZFNs and TALENs recognize target sequences by protein motifs, which requires researchers to assemble specific proteins for each target and limits their widespread adoption (Gaj et al., 2013). In contrast, the CRISPR/Cas system recognizes genomic target sites by base complementary pairing between the single-guide RNA (sgRNA) and the target DNA, which greatly simplifies its application (Xie and Yang, 2013). Many miRNAs have been characterized by TALENs in animals, but only one study has made TALEN constructs for miRNA gene editing that resulted in heritable mutations in plants (Bi et al., 2020). The CRISPR/Cas9 system was successfully used to conduct protein-coding gene editing in *Arabidopsis* (Li et al., 2013), *Nicotiana benthamiana* (Nekrasov et al., 2013), and rice (Shan et al., 2013) soon after its establishment. Recently, many miRNAs have been successfully knocked out in various plant species by the introduction of indels at *MIR* genes *via* CRISPR/Cas9 nonhomologous end joining (NHEJ; Damodharan et al., 2018; Hou et al., 2019; Miao et al., 2019). In addition, the full-length deletion and knock-in of *MIR* genes can also be achieved by CRISPR/Cas9 editing *via* homology-directed repair (HDR; Zhao et al., 2016). Moreover, the CRISPR/Cas9 system can be used to modulate gene expression through the transcriptional activation or repression of target genes by fusing a deactivated Cas9 nuclease (dCas9) with transcriptional regulators such as transcriptional activators (Li et al., 2017) and repressors (Tang et al., 2017). Although the CRISPR/Cas9 system is an efficient tool to modify the sequence of miRNA genes and generate miRNA null mutant plants, the short length of *MIR* genes limits the design of gRNA targets that target *MIR* genes. In addition, multiple miRNA family members in the genome may limit the application of CRISPR/Cas9 in knocking out the whole *MIR* gene family simultaneously.

#### Techniques Targeting Pri-/Pre-miRNAs

Various techniques that are commonly applied to silence protein-coding genes by targeting the transcripts have been adopted for functional analyses of miRNAs (Figure 2C). RNAi, widely used for the knockdown of coding genes, is also able to suppress the accumulation of miRNAs. MiR163 and miR171a were successfully blocked by RNAi constructs designed to target both the primary miRNA transcripts and their promoters (Vaistij et al., 2010). Through RNAi, a diverse set of siRNAs are produced that might potentially trigger off-target effects. To avoid off-target effects, an amiRNA strategy was developed to specifically silence targets by expressing amiRNA with miRNA precursors as backbones (Carbonell et al., 2016). By replacing the original miRNA/miRNA* with amiRNA/amiRNA* designed to target a specific mRNA, genes of interest can be successfully blocked (Warthmann et al., 2008). It has been reported that all family members can be silenced by an amiRNA designed to target the mature sequence of a miRNA, in contrast, only the individual member can be silenced by an amiRNA designed to target the nonconserved stem-loop region of the precursor transcript, which benefits the functional validation of individual *MIR* loci in the genome (Eamens et al., 2011). To silence targets of interest effectively and specifically, the selection of both amiRNA and pre-amiRNA sequences should be accurate and suitable, this is the main challenging task associated with the utilization of amiRNA (Carbonell et al., 2016; Peng et al., 2018).

Furthermore, with class II type VI-A endoribonuclease, CRISPR/Cas13a can target and cleave single-stranded RNA guided by gRNA, which can also be used to target pri-/pre-miRNA transcripts of *MIR* genes (Aman et al., 2018). However, the CRISPR/Cas13a system has not yet been tested for miRNA silencing in plants.

#### Techniques Targeting Mature miRNAs

Mature miRNAs can be decoyed by exogenous synthetic AMOs and endogenous target mimics (Figure 2D). AMOs are chemically modified antisense oligonucleotides designed to pair with and block mature microRNAs by sequence complementarity, this approach was initially used in animals to suppress miRNA activity (Hutvágner et al., 2004; Meister et al., 2004). In the recent years, it has been reported that AMO can induce the efficient inhibition of miRNAs by sucrose-mediated delivery in rice protoplasts and intact leaves (He et al., 2016). AMOs function in a sequence-specific manner against targeted miRNAs and transiently induce miRNA blockages, which enables a quick assessment for the characterization of miRNAs in plants. A study by Franco-Zorrilla et al. (2007) demonstrated that endogenous target mimics could block the interaction between miRNAs and their targets, thereby silencing miRNA function. In *Arabidopsis*, *INDUCED BY PHOSPHATE STARVATION 1* (*IPS1*), an endogenous long noncoding RNA, was found to have a miR399-binding site with a central “bulge” formed by three nucleotides (CUA) that could effectively trap miR399 and abolish miR399-guided cleavage (Franco-Zorrilla et al., 2007). In plants, miRNAs were decoyed by replacing the endogenous miR399 target site of *IPS1* with artificial TMs of interest (Todesco et al., 2010; Sun et al., 2020). In animal cells, four copies of the miRNA-binding sites with two central mismatches at the cleavage site linked by 4-nt spacers were used to inhibit miRNA action in SP systems (Ebert et al., 2007). In recent years, SPs have been successfully applied to block miRNAs in plants (Reichel et al., 2015; Tong et al., 2017; Beltramino et al., 2018). More miRNA-binding sites (up to 15) are used in a sponge to increase its efficacy in inhibiting miRNA action in plants. Nevertheless, it is difficult to construct a long SP with multiple tandem repeats, which may limit the application of SPs. STTM was initially developed based on the principle of TMs and SPs. STTM is a modified artificial RNA structure containing two miRNA-binding sites linked by an RNA spacer of 48–88nt. Like those of TMs, miRNA-binding sites of STTMs have mismatches at the miRNA cleavage sites, which help STTMs sequester miRNAs without being cleaved by them. The spacer between the two miRNA-binding sites forms a mild “stem,” which serves as an optimal structure important for both preventing Dicer attack and stabilizing the expressed STTM in cells (Yan et al., 2012). STTMs can block or destroy specific endogenous small RNA functions in plants and effectively knock down the expression of miRNAs of an entire family (Yan et al., 2012). MiRNA degradation triggered by STTM is partly dependent on SDN-mediated miRNA degradation, but the mechanism is not fully understood (Yan et al., 2012; Teotia et al., 2016). In addition, the F-box protein HAWAIIAN SKIRT (HWS) plays a critical role in miRNA degradation triggered by TM/STTM system, which may result from its function in the clearance of non-optimal RISC induced by mimicry target (Lang et al., 2018; Mei et al., 2019). Perturbing the function of miRNAs as miRNA decoys, it has been reported that the silencing efficacy of TMs, SPs, and STTMs varies among different miRNA families. STTMs have been verified to be effective in perturbing activities of highly abundant miRNAs (e.g., miR165/166), TM more effectively targets low-abundance miRNAs (e.g., miR159), and SPs function as an alternative for miRNAs that cannot be effectively blocked by either TM or STTM (Reichel et al., 2015). Thus, multiple decoy strategies are recommended in order to generate the desired outcome.

#### Techniques Mimicking the Loss of Function of miRNAs

Another approach to mimicking the loss-of-function state of a miRNA is upregulating the targets of miRNAs by overexpressing the cleavage-resistant target genes (Li and Millar, 2013; Figure 2A). Since overexpressed original targets can still be cleaved by miRNAs, cleavage-resistant targets are generated by modifying the miRNA cleavage site by creating synonymous mutations. Many transgenic plants overexpressing cleavage-resistant targets have been created, such as *Arabidopsis* expressing miR156-resistant *AtSPL3* (*rSPL3*; Kim et al., 2012) and the miR172-resistant form of *AtTOE3* (Jung et al., 2014), tomato expressing miR164-resistant *SlNAM2* (Hendelman et al., 2013), and rice expressing miR319-resistant *OsTCP21* (*rTCP21*; Zhang et al., 2016a) and miR166-resistant *OsHB4* (Zhang et al., 2018b). This approach could be an additional way to investigate the functions of the corresponding miRNAs.

## STTM Techniques in Plant Research

**Figure 3:**
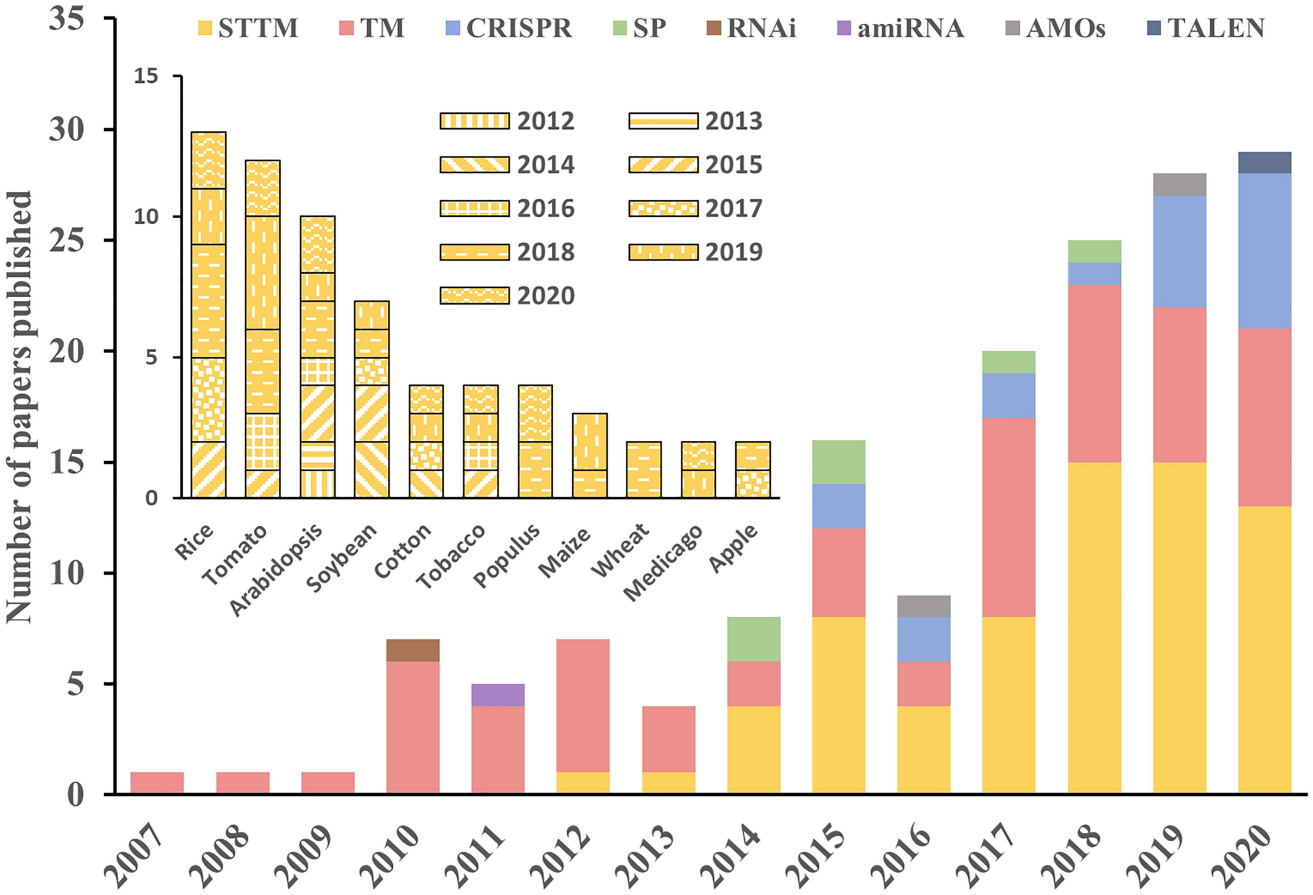
Applications of different approaches in the literature. The histograms show the number of manuscripts published per year using the different approaches for miRNA study in plants (outer) and the application of STTM in different species (inner). The information used in this figure was retrieved through April 30, 2021, from PubMed.

A gain-of-function system that constitutively overexpresses a miRNA may alter its localization and dose. Therefore, the non-authentic phenotypes may not reflect the normal function of the miRNA. Thus, various loss-of-function strategies have been extensively exploited as alternative and effective approaches to evaluate the roles of many miRNAs. Of these approaches, miRNA decoy techniques such as TM and STTM, and the genome-editing system CRISPR/Cas9 are applied much more widely than others (Figure 3). As the first miRNA decoy technique created for plants, the TM approach has been applied in many studies, and the functions of many miRNAs have been successfully uncovered (Todesco et al., 2010). In recent years, STTM and CRISPR/Cas9 techniques have been developed rapidly and have become the two most widely adopted approaches in functional analysis of miRNAs in plants.

The STTM technique is widely accepted in miRNA functional studies in plants because it can be applied in a variety of ways. First, the STTM structure has two miRNA-binding sites, and it can efficiently silence some highly abundant miRNAs (e.g., miR156/157, miR165/166, and miR398) and generate visible phenotypes (Yan et al., 2012; Reichel et al., 2015; Zhang et al., 2017). Second, STTMs can be used to study the interactions between two miRNAs by inserting two different miRNAs in the same STTM construct (Peng et al., 2018). Moreover, it has been reported that STTMs can silence multiple distinct miRNAs simultaneously with an increased number of tandem miRNA-binding sites (Fei et al., 2015). Finally, the STTM approach can be adopted as an important complement to CRISPR/Cas9 in certain situations, such as the knockout of miRNAs resulting in drastic or lethal developmental defects (Teotia et al., 2016) or the locations of miRNAs overlapping with other genes in the chromosome.

The STTM approach can be applied in a constitutive (Tang et al., 2012), inducible (Peng et al., 2018) or tissue-specific (Zhao et al., 2019) manner driven by the corresponding promoter. This makes it possible to study miRNA functions spatiotemporally and to precisely modify the crop traits of interest. Since its development, the STTM approach has been extensively adopted for the functional analysis of numerous miRNAs in multiple species, including rice (Xia et al., 2015; Zhang et al., 2017; Zhao et al., 2019), tomato (Damodharan et al., 2016; Jiang et al., 2018; Yang et al., 2020), soybean (Wang et al., 2014; Nizampatnam et al., 2015), cotton (Wang et al., 2017; Liu et al., 2019), maize (Zhang et al., 2019), wheat (Guo et al., 2018), and tobacco (Diao et al., 2019; Figure 3). Rice is the most important staple crop worldwide, and several of its agriculturally important traits are controlled by miRNAs (Tang and Chu, 2017; Peng et al., 2019). Given the extensive application of STTMs in rice, we reviewed STTM-based functional studies of miRNAs in rice and focused specially on agronomic traits.

## Application of the Sttm Technique for Rice Functional Genomics and Breeding

**Figure 4:**
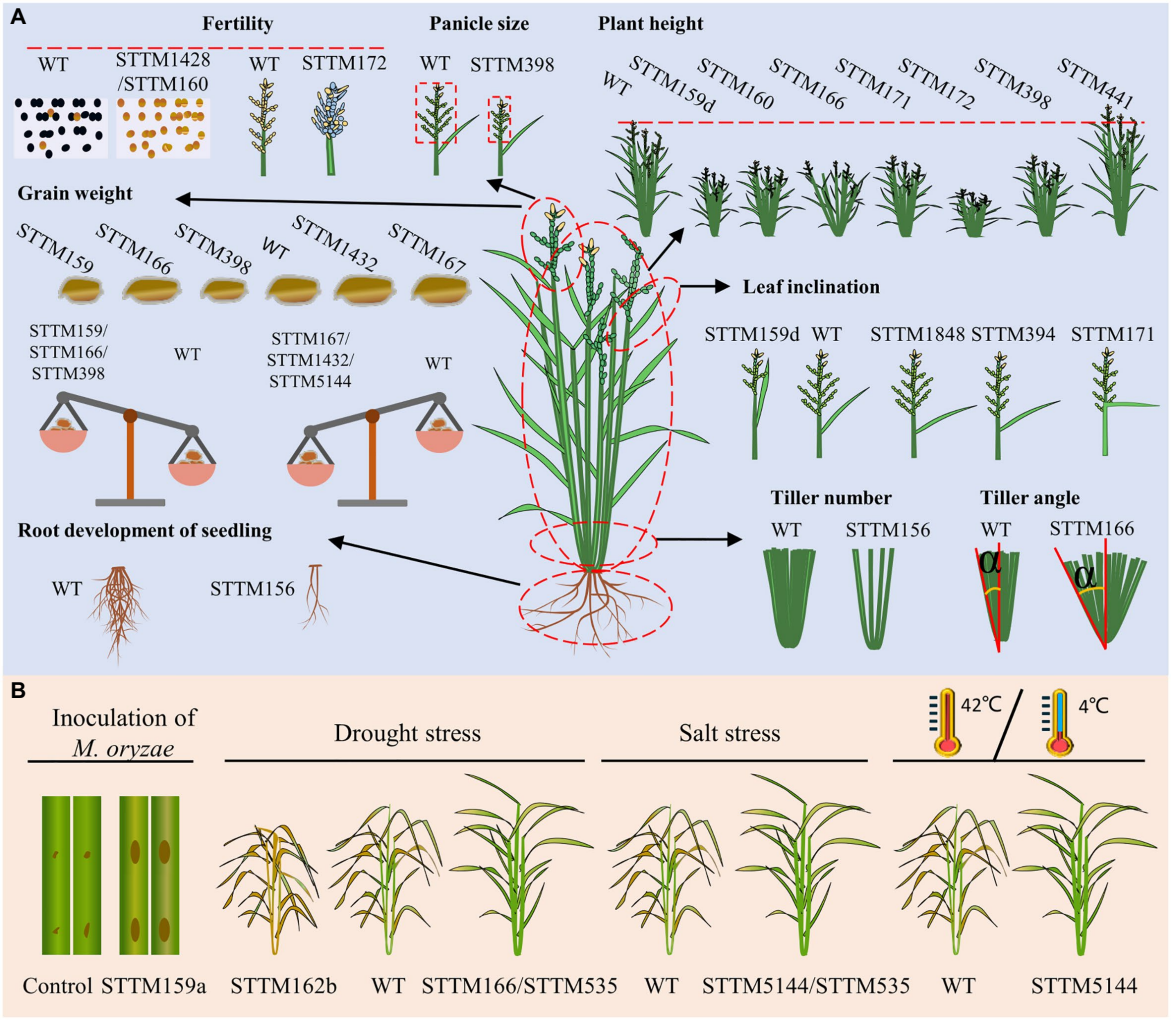
Experimentally verified phenotypes of rice STTM lines: agronomic trait-related **(A)** and stress response-related **(B)**. **(A)** Variations in agronomic traits, including the fertility, panicle size, plant height, grain weight, leaf inclination, tiller number, tiller angle, and root development, of seedlings are diagrammatized. Fertility: fertile (black dots), sterile (yellow dots), filled seeds (yellow grains), empty seeds (blue grains). Grain weight: smaller grains (left side of WT), larger grains (right side of WT), each part of the balance represents the 1,000-grain weight. Leaf inclination: smaller leaf angle (left side of WT), increased leaf angle (right side of WT). **(B)** Diagrams from left to right represent biotic stress from blast disease caused by *Magnaporthe oryzae* and abiotic stress, including drought stress, salt stress, high temperature (42°C), and low temperature (4°C). Brown dots on the leaves indicate disease lesions, deep yellow plants indicate dead tissues and green plants indicate surviving tissues.

Higher yield and enhanced stress resistance are important breeding goals of crop breeders. Yield is a sophisticated agronomic trait in rice that is generally determined by four decisive factors: the number of tillers per plant, number of grains per panicle, grain size/weight, and the ratio of filled grains (Sakamoto and Matsuoka, 2008; Xing and Zhang, 2010). In addition, tiller angle and plant height are important agronomic traits that determine the ideal architecture and the leaf size, shape, and inclination determine the leaf architecture, all of which eventually affect the grain yield (Zuo and Li, 2014). Extensive studies have supported the vital roles of miRNAs in regulating diverse important agronomic traits and stress responses in crops (Zheng and Qu, 2015; Tang and Chu, 2017; Peng et al., 2019). To date, dozens of miRNA families have obtained STTM transgenic lines in rice, and several of them show obvious phenotypic alterations associated with yield-related agronomic traits and stress responses (Figure 4; Table 1). Hereafter, we provide an overview of the application of STTM in rice classified by different agronomic traits.

**Table 1:** Summary of functionally validated miRNAs by STTM in rice.

Agronomic traits		STTM-miRNA lines	Promoter used	Reference
Fertility	Sterile pollen	miR160, miR1428	35S	Zhang et al., 2017
	Decreased seed setting rate	miR172		
Grain weight	Decreased grain weight	miR156, miR159, miR165/166, miR398	35S	Zhang et al., 2017; Zhao et al., 2017; Gao et al., 2018; Zhang et al., 2018b
	Increased grain weight	miR167, miR1432	Gt13a	Peng et al., 2018; Zhao et al., 2019
		miR5144-3p	Ubi-1	
Panicle	Shorter panicle	miR159, miR171, miR398	35S	Zhang et al., 2017; Zhao et al., 2017
	Semi-enclosed panicle	miR159, miR171	35S	Zhang et al., 2017; Gao et al., 2018
Plant height	Decreased plant height	miR159, miR165/166, miR171, miR172, miR398	35S	Zhang et al., 2017; Zhao et al., 2017; Gao et al., 2018; Zhang et al., 2018b; Chen et al., 2021a
	Increased plant height	miR441	35S	Zhang et al., 2017
Leaf inclination	Decreased leaf inclination	miR159	35S	Gao et al., 2018
	Enhanced leaf inclination	miR171, miR394	35S	Xia et al., 2015; Zhang et al., 2017; Qu et al., 2019
		miR1848	Ubi-1	
Tiller number	Fewer tillers	miR156	35S	Zhang et al., 2017
Tiller angle	Wider tiller angle	miR165/166	35S	
Root	Shorter roots with reduced root number	miR156	35S	
Abiotic stress	Enhanced susceptibility to *M. oryzae*	miR159	35S	Chen et al., 2021b
Drought stress	Reduced tolerance	miR162b	Ubi-1	Tian et al., 2015
	Enhanced tolerance	miR166	35S	Zhang et al., 2018b
		miR535	Ubi-1	Yue et al., 2020
Salt stress	Enhanced tolerance	miR5144-3p, miR535	Ubi-1	Xia et al., 2017; Yue et al., 2020
High/low temperature	Enhanced tolerance	miR5144-3p	Ubi-1	Xia et al., 2017

### Tiller Number Related miRNA Confirmed by the STTM Approach

The tiller number per plant is one of the factors that contribute to rice yield, and several miRNA-target modules have been reported to play roles in regulating the tiller number per plant (Peng et al., 2019). It is well characterized that miR156 functions in rice tiller formation by cleaving its target *OsSPL14* in the vegetative stage (Jiao et al., 2010; Miura et al., 2010). Transgenic lines overexpressing miR156 showed increased tiller numbers per plant (Xie et al., 2006; Hayashi-Tsugane et al., 2015; Liu et al., 2015). The knockdown of miR156 by the STTM approach revealed additional roles in the control of shoot and root development at the seedling stage (Zhang et al., 2017).

### Grain Number-Related miRNAs Confirmed by the STTM Approach

The grain numbers per panicle are determined by the panicle architecture: branches, panicle axis, and spikelets. It was reported that *Arabidopsis* miR398 plays role in various stresses by targeting Cu/Zn-superoxide dismutases (*CSD1* and *CSD2*; Zhu et al., 2011), and no obvious developmental or architectural phenotypes have been revealed. In rice, silencing miR398 by the STTM approach led to reduced grain numbers per panicle because of shorter and smaller panicles (Zhang et al., 2017), suggesting its significant function in panicle growth.

### Grain Weight-Related miRNAs Confirmed by the STTM Approach

Grain weight is determined by grain length, width, and thickness, the grain-filling rate and grain-filling period. STTM156 plants had slightly increased grain length and 1,000-grain weight. STTM159 lines in rice showed smaller grains and lower grain weight through its target *OsGAMYBL2* (Zhang et al., 2017; Zhao et al., 2017). In addition, OsmiR159d STTM lines specifically blocking the functions of miR159d also had smaller grains (Gao et al., 2018). STTM166 lines showed decreased rice grain weight with decreased grain width but increased grain length (Zhang et al., 2018b). STTM167 transgenic *Arabidopsis* often shows incompletely filled seeds. Rice STTM167 driven by the Gt13a endosperm-specific promoter showed a substantial increase in the grain weight, indicating that miR167 regulates seed development oppositely in monocot and dicot plants (Peng et al., 2018). Blocking miR398 by the STTM approach also results in significantly decreased grain length and width, causing a 40% decrease in 1,000-grain weight (Zhang et al., 2017). STTM1432 plants showed a substantial increase (46.69%) in grain weight due to an improved grain filling rate, which was photocopied by overexpression of miR1432-resistant *OsACOT* (Zhao et al., 2019). STTM5144-3p plants produce grains with increased 1,000-grain weight, which was also the result of the overexpression of its target *OsPDIL1;1* (Xia et al., 2017).

### Seed Setting Rate-Related miRNAs Confirmed by the STTM Approach

Seed-setting rate relies strongly on seed setting after successful double fertilization and starch accumulation. Knockdown of miR172 by the STTM approach resulted in a significantly decreased seed-setting rate (Zhang et al., 2017). The lower seed-setting rate of STTM172 plants was presumably resulted from the enclosed panicle since normal fertile pollens were produced. In addition, the STTM160 and STTM1428 lines were reported to show dramatically decreased seed-setting rates because of complete male sterility in rice (Zhang et al., 2017). Overall, the roles of miRNAs in the regulation of fertility and panicle morphology contribute to the seed-setting rate.

### Plant Architecture-Related miRNAs Confirmed by the STTM Approach

In addition to the tiller number, the tiller angle, leaf inclination, and plant height are important agronomic traits that contribute to ideal plant architecture and grain production (Sakamoto and Matsuoka, 2008; Zuo and Li, 2014; Mantilla-Perez and Salas Fernandez, 2017). STTM166 lines showed an increased tiller angle, but the underlying regulatory mechanism of this is not yet clear (Zhang et al., 2017). MiR1848-*OsCYP51G3* module determines leaf inclination by affecting BR biosynthesis. STTM1848- and *OsCYP51G3*-overexpressing transgenic lines showed a larger leaf inclination (Xia et al., 2015). An auxin-responsive module, miR394-*OsLC4*, functions in determining rice leaf inclination through auxin homeostasis. STTM394 lines showed greatly enhanced leaf inclination and altered auxin responses (Qu et al., 2019). STTM159 lines showed reduced stature and stem diameter (Zhang et al., 2017; Zhao et al., 2017). The silencing of miR160 by STTM in rice reduced plant height, similar to the results in *Arabidopsis*, indicating its conserved function in plant height between monocots and dicots (Zhang et al., 2017). STTM166 lines exhibited reduced plant height resulting from decreased length of internodes by targeting *OsHB4*, a member of HD-Zip III gene family (Teotia et al., 2017; Zhang et al., 2017). In addition, miR166b was reported to regulate cell wall biosynthesis by targeting *OsHox32*. The knockdown of miR166b by STTM resulted in droopy leaves and brittle culms due to reduced cell wall thickness with decreased accumulation of lignin and cellulose (Chen et al., 2021a). Knockdown of miR171 by STTM resulted in semi-dwarf plants, semi-enclosed panicles, and droopy flag leaves (Zhang et al., 2017). STTM172 lines showed unexpected phenotypes in rice, dwarf plants resulted from shorter culms compared with wild-type plants (Zhang et al., 2017). MiR441 was identified to increase plant height after knockdown by STTM in rice, but the underlying mechanism is not clear yet (Zhang et al., 2017).

### Stress Response-Related miRNAs Confirmed by the STTM Approach

In addition to their involvement in agronomic traits observed under natural paddy field conditions, several STTM-miRNA transgenic lines showed altered responses to biotic and abiotic stresses (Figure 4B; Table 1). MiR159a is a positive regulator of resistance to *Magnaporthe oryzae* (*M. oryzae*) by targeting *OsGAMYB*, *OsGAMYBL*, and *OsZF*. STTM159a lines showed enhanced susceptibility (Chen et al., 2021b). MiR162b, miR166, and miR535 were confirmed to participate in the response to drought stress. STTM162b lines showed a greatly decreased survival rate compared to WT, and miR162b was identified as a positive regulator of drought resistance by targeting *OsTRE1* (Tian et al., 2015). MiR166 modulates the morphology of leaves and the size of xylem vessels by targeting *OsHB4*. STTM166 lines showed enhanced drought resistance resulting from decreased transpiration rates and hydraulic conductivity due to reduced stomatal conductance and xylem vessel diameter, respectively (Zhang et al., 2018b). STTM535 lines showed enhanced resistance to drought and salinity, and *OsSPL19* is presumed to be the main functional target of miR535 in response to drought and salinity (Yue et al., 2020). MiR5144-*OsPDIL1;1* module modulates the formation of protein disulfide bonds, which affects the correct folding of proteins and is important for protein function in the stress response. STTM5144- or *OsPDIL1;1*-overexpressing lines showed enhanced resistance to various abiotic stresses including HgCl_2_, salinity, high temperature, and low temperature, due to the increased content of protein–disulfide bonds (Xia et al., 2017). To date, only a small portion of STTM lines have been evaluated under stress conditions. All these STTM transgenic lines generate a resource pool that contributes to mining novel targets for agronomic improvement by assessing their responses to biotic and abiotic stresses.

## Applications of the CRISPR/Cas9 System in Transgene-Free Crop Breeding

Although many agronomically improved STTM transgenic lines have been generated, these achievements have not yet been commercialized. Currently, the commercial development of genetically modified organisms (GMOs) is considered a threat to human health and the environment and is thus under stringent governmental regulations (Prado et al., 2014). Transgene-free edited crops created *via* CRISPR/Cas9 receive the regulatory waivers in many countries (Chen and Gao, 2020). Transgene-free edited crops can be generated through conventional and transient expression methods. In conventional transformation methods, transgene-free edited progenies can be obtained by eliminating CRISPR transgenes *via* genetic segregation through selfing or crossing (Gao, 2021). In the transient expression methods, transgene-free genome editing can be generated through the transient expression of CRISPR/Cas9 DNA, RNA, or RNP (ribonucleoprotein) delivered into plant cells (Woo et al., 2015; Zhang et al., 2016b; Liang et al., 2017). Recently, transgene-free gene-edited non-browning white button mushrooms (Waltz, 2016) and high-GABA tomato (EUROFRUIT, 2021) have been commercialized outside of GMO regulations. Furthermore, due to the precise genome modifications, time savings, and transgene-free nature of these crops (Zhu et al., 2020), some practices for translating the outcomes of transgenic into transgene-free breeding by CRISPR/Cas9 in crops have been developed.

Great progress has been made by using a transgene-free edited system on protein-coding genes. A series of transgene-free edited crops with increased yield (Li et al., 2016), improved quality with low amylose content (Zhang et al., 2018a), high amylose content (Sun et al., 2017; Tuncel et al., 2019; Li et al., 2021), higher grain fragrance (Usman et al., 2020), enriched γ-aminobutyric acid (Akama et al., 2020), and enhanced biotic stress resistance (Zhou et al., 2020) have been generated. Currently, several studies are also trying to convert the accumulating knowledge of miRNA functions into a transgene-free edited system. In rice, the overexpression of *OsmiR535* resulted in reduced tolerance to drought and salinity, while the knockdown of *OsmiR535* by STTM conferred enhanced tolerance to drought and salinity (Yue et al., 2020). A transgene-free, drought-tolerant *osmir535* mutant was generated by CRISPR/Cas9 mediated knockout, which provides a successful example for abiotic stress-resistant breeding (Yue et al., 2020). In soybean (*Glycine max* L.), gma-miR398c functions negatively in drought tolerance by targeting multiple peroxisome-related genes, *GmCSDs* and *GmCCS* (Zhou et al., 2020). *Arabidopsis* and soybean overexpressing gma-miR398c both showed decreased drought tolerance, while the *gma-miR398c* mutant generated by CRISPR/Cas9 showed increased drought resistance. Gma-miR398c was demonstrated to be a valuable locus for crop improvement by creating drought resistance-enhanced soybean *via* CRISPR/Cas9 (Zhou et al., 2020). In tomato, one of the most important horticultural crops, late blight caused by *Phytophthora infestans* (*P. infestans*) is a great threat to production (Fry et al., 2015). MiR482 was identified as a negative regulator of the resistance to *P. infestans* by targeting *NBS–LRR* disease-resistance genes in tomato (Jiang et al., 2018; Canto-Pastor et al., 2019). The overexpression of miR482b caused more severe disease symptoms in plants infected by *P. infestans*, while the silencing of miR482b by STTM resulted in enhanced resistance to *P. infestans* in tomato (Jiang et al., 2018). Based on these transgenic outcomes, *mir482b* and *mir482b/c* were generated by CRISPR/Cas9, and both showed enhanced resistance to *P. infestans*. In addition, *mir482b/c* was more resistant to *P. infestans* than *mir482b* in tomato, demonstrating that miR482 is an important target for cultivating pathogen-resistant tomatoes *via* CRISPR/Cas9 (Hong et al., 2020). The ever-expanding genetic resources created by these miRNA techniques provide us with a promising prospect for crop improvement and breeding.

## Conclusion and Perspectives

Multiple tools have been developed for the functional analysis of miRNAs, thus contributing to the crop improvement and future agriculture. Given the advantages and drawbacks of each approach, it is important to design proper strategies for each specific study and the miRNAs of interest. STTM is one of the most widely adopted miRNA techniques, and a large collection of STTM lines have been generated in crops, especially in rice. STTM and related miRNA techniques help to reveal the complex molecular mechanisms involved in the regulation of agronomic traits, and also provide potential breeding materials for crop improvement and breeding. These transgenic achievements, together with the CRISPR/Cas gene-editing system and many other breeding techniques, will allow the generation of transgene-free plants and greatly facilitate precision crop breeding.

To date, as the majority of functional studies focus on conserved miRNAs, our knowledge about species and tissue specific miRNAs is extremely limited, which offers a broad space for the application of STTM and other approaches. Further identification and validation of these miRNAs will lead to a great leap for the application of miRNAs in crop improvement. Due to the multiple roles of miRNAs in plant developmental processes, STTM lines of these miRNAs driven by the 35S promoter show pleiotropic effects that alter more than one trait (Table 1). Tissue-specific or inducible promoters may be an appropriate alternative to facilitate STTM as a useful tool to study the functions of miRNAs in specific tissues and increase yield without penalty on other agronomic traits. Not all STTM rice lines showed apparently altered morphologies (Zhang et al., 2017), and other approaches mentioned previously are advocated to be adopted for the desired loss-of-function outcome. Multiple genetics-based approaches are imperative and may work complementarily in fully mining and using vital miRNAs associated with superior agronomic traits. MiRNAs are important regulators in biotic and abiotic stress responses. Fine-tuning the accumulation of stress-related miRNAs by STTM techniques may provide valuable resources for the assessment of varying stress responses. Additionally, due to public safety concerns over transgenic crops, the commercialization of improved crops bred by STTM-based techniques is restricted. Some successful breeding practices on translation of transgenic outcomes into transgene-free outcomes *via* CRISPR/Cas9 in rice have been adopted. Useful targets for crop improvement confirmed by STTM have huge potential for application in breeding through non-transgenic translation, which may accelerate the creation of new varieties and strengthen food security.

## Author Contributions

GT supervised the project. JC and TL wrote this paper. ST edited and gave suggestions for the manuscript. All authors contributed to manuscript revision, read, and approved the submitted version.

## Funding

This work was supported by funding from the Guangdong Innovation Research Team Fund (grant no. 2014ZT05S078), the National Natural Science Foundation of China (grant no. 32070852) and the open fund of the Guangdong Key Laboratory of Plant Epigenetics.

## Conflict of Interest

The authors declare that the research was conducted in the absence of any commercial or financial relationships that could be construed as a potential conflict of interest.

## Publisher’s Note

All claims expressed in this article are solely those of the authors and do not necessarily represent those of their affiliated organizations, or those of the publisher, the editors and the reviewers. Any product that may be evaluated in this article, or claim that may be made by its manufacturer, is not guaranteed or endorsed by the publisher.
